# Editorial: The etiology and pathogenesis of affective disorders

**DOI:** 10.3389/fpsyg.2023.1233256

**Published:** 2023-07-18

**Authors:** Jiabao Lin

**Affiliations:** Department of Applied Psychology, School of Public Health and Management, Guangzhou University of Chinese Medicine, Guangzhou, Guangdong Province, China

**Keywords:** affective disorders, anxiety disorders, depression disorders, social anxiety disorder, mechanisms

Affective disorders have already become a concerning issue around the globe. The World Health Organization (WHO) has conducted surveys and found that affective illnesses may result in disability and lower life quality to a great extent by 2030, which can correspondingly bring great financial burdens to the public health system. Typical affective disorders contain a series of mental diseases like anxiety disorders, depression disorders and bipolar disorders. They can simultaneously and negatively affect one's emotional feelings and cognitive functions, where they may gradually fall into worse conditions (e.g., self-hurt behavior). Given probable severe consequences, it turns out to be an urgent need to better understand what can lead to affective disorders, which may provide viable intervention targets for the treatment or prevention.

Specifically, anxiety is one of the most commonly seen affective disorders that should have attached more importance to it. Anxiety plays an early warning role to arise vigilance in the alert system. Anxiety can produce a powerful influence that keeps penetrating our life, especially when facing potentially threatening circumstances. However, what should be noticed is that anxiety is more than a unidimensional concept. It is made up of several subtypes when it comes to specific classification and identification of diagnosis in DSM-5, such as generalized anxiety disorder (GAD), panic disorder (PD) and social anxiety disorder (SAD). These phobic disorders are frequently characterized by fear, emotion and safety behavior (i.e., impression management, avoidance behavior and anxiety-symptom control). But patients suffering from SAD tend to have an explicit source (i.e., social situations) provoking fear with high intensity automatically. They might be extremely shy during impersonal communication, avoiding eye contact with others and sometimes feeling too afraid to express their opinions. Individuals with SAD can't help getting trapped in a vicious circle easily, with low self-esteem, while self-doubt and criticism are high. They may find it hard to regulate their overreaction emotionally and behaviorally. Much evidence did show that SAD can lead to functional impairments and disability. It is associated with a higher possibility of dropping out of school, work absence, low-quality life, substance addiction and even suicidal activity.

Regarding the corresponding mechanisms behind SAD, it is necessary to conceive a complete picture with the help of pathophysiology and psychological discoveries. Existing clinical research has evidenced that abnormalities detected in the neurotransmitting system and hypothalamic-pituitary-adrenal axis could be the probable cause of inducing SAD. A handful of substances like dopamine, GABA, serotonin and norepinephrine, whose regular secretion is compulsory for maintaining inner homeostasis and supporting ordinary outer activities, were reported to reach an aberrant level. Besides, genetics studies suggest the familial influence as well. Family profiles of severe mental diseases are commonly seen as contributive factors of SAD found in the younger generation. Additionally, a related neuroimaging system review (Mizzi et al., 2022) deserves extra attention since they also depicted abnormal resting-state brainy patterns between the SAD and healthy individuals. Researchers assembled large-scale neuroscientific data and figured out the dissimilar connectivity signals between frontal–amygdala and frontal–parietal regions in SAD patients instead of the health control ones. This outcome can imply dysfunctions of SAD, which are exactly roots in neural alternation. However, all these mentioned pathophysiological causes can only explain the phenomenon partially. What helps to catalyze the final occurrence of the affective disorder are certain personality traits. One study, Soodla and Akkermann suggested that personal features like high neuroticism and low extraversion should be highlighted to be more concrete. And such characteristics can be traced back to negative parenting styles and childhood maltreatment. Adults with SAD can more easily recall the overprotective and controlling ways executed by their parents in their early life. At the same time, traumatized experiences in childhood like emotional abuse, emotional neglect and physical neglect are the risk factors to efficiently predict the severity of SAD symptoms.

Except for the subtypes within the spectrum of anxiety disorders, comorbid psychiatric symptoms or severe mood disorders also need more attention. The fact that comorbid anxiety and depression are universal in clinical practice. Previous evidence from Japan (Noda et al.) showed that SAD could significantly predict the first onset of depression. The rejection sensitivity symptom (i.e., overreaction to rejection), which is one of core SAD features, could lead to troublesome pathological states and reach severe depression finally. SAD patients suffering from a major depressive disorder at the same time exhibited an increased risk of suicidality and other chronic diseases. They could lose the ability to regulate their emotions, have excessive rumination about others' intentions and set up negative expectations toward potential outcomes. This “over-mentalizing” activity was supposed to be linked with prefrontal activity at the brain level.

At present, numerous research does attempt to depict the potentially-unknown mechanisms behind mood disorders with the help of different types of data. All those examinations and verification hint that patients with SAD possibly differ physiologically and psychologically from the healthy. Corresponding interventions should not be limited to pure medication treatment or psychological counseling. Instead, it is necessary to consider these two ways comprehensively.

## Author contributions

JL contributed to the conception of the manuscript, drafting the manuscript, providing revisions, and approved the final version of the manuscript for submission.
